# The Genome as an Evolutionary Timepiece

**DOI:** 10.1093/gbe/evw220

**Published:** 2016-09-07

**Authors:** Simon Y. W. Ho, Amanda X. Y. Chen, Luana S. F. Lins, David A. Duchêne, Nathan Lo

**Affiliations:** School of Life and Environmental Sciences, University of Sydney, Sydney, NSW, Australia

**Keywords:** molecular clock, molecular dating, evolutionary rate, genomic data, phylogenetic analysis

## Abstract

The molecular clock is a valuable and widely used tool for estimating evolutionary rates and timescales in biological research. There has been considerable progress in the theory and practice of molecular clocks over the past five decades. Although the idea of a molecular clock was originally put forward in the context of protein evolution and advanced using various biochemical techniques, it is now primarily applied to analyses of DNA sequences. An interesting but very underappreciated aspect of molecular clocks is that they can be based on genetic data other than DNA or protein sequences. For example, evolutionary timescales can be estimated using microsatellites, protein folds, and even the extent of recombination. These genome features hold great potential for molecular dating, particularly in cases where nucleotide sequences might be uninformative or unreliable. Here we present an outline of the different genetic data types that have been used for molecular dating, and we describe the features that good molecular clocks should possess. We hope that our article inspires further work on the genome as an evolutionary timepiece.

## Introduction

Estimates of evolutionary rates and timescales form an important component of research in biology. These can be obtained from genomes using molecular clocks, which describe the relationship between genetic change and geological time. The idea of a molecular clock was first put forward >50 years ago. In their seminal study, Zuckerkandl and Pauling (1962) estimated the rate of haemoglobin evolution based on the amino acid differences between human and horse, which diverged ∼100–160 Ma. By assuming that this rate has remained constant across lineages, they inferred divergence times for several different haemoglobin genes and between human and gorilla.

With major advances in DNA-sequencing methods from the 1980s, the use of amino acid sequences in molecular dating has declined substantially. Nevertheless, they are still often employed in studies of deep timescales, particularly when nucleotide sequences exhibit high degrees of saturation or variability in nucleotide composition (dos Reis et al. 2015; Lozano-Fernandez et al. 2016). Nucleotide sequences are now the dominant form of genetic data, with a growing number of molecular-clock studies using genome-scale data sets (Jarvis et al. 2014; Misof et al. 2014). These data have allowed detailed studies of evolutionary rate variation and have spurred a considerable amount of methodological development (Ho 2014; Donoghue and Yang 2016). However, there is much more to the genome than just sequence data. Genomes offer a rich source of information for estimating evolutionary timescales, but this potential has remained largely untapped. Here we discuss the outlook for different types of molecular clock, providing insight into the genome as an evolutionary timepiece.

## What Makes a Good Molecular Clock?

**Fig. 1.—:**
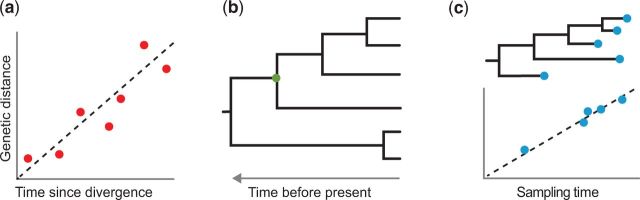
Methods used to estimate evolutionary timescales from genomic data. (*a*) Linear regression of pairwise genetic distances against time since divergence. Each data point in this plot represents a pair of taxa, with their divergence time inferred from the fossil record or from the age of a geological event that is presumed to be associated with the evolutionary divergence. Fitting a line through these points involves the assumption that genetic change accumulates at a constant rate through time, with the slope presenting an estimate of this rate. The line of best fit can be used to infer the timing of evolutionary divergence events, provided that a measure of genetic distance is available for the taxa in question. Molecular clocks based on linear regression have a number of weaknesses, including nonindependence of the data points and sensitivity to rate variation across lineages. (*b*) Phylogenetic analysis using a clock model. The tree is a chronogram with branch lengths measured in units of time. These methods usually involve models that explicitly describe the evolution of characters along the branches of the tree. Phylogenetic molecular clocks are calibrated by constraining the age of one or more nodes in the tree, such as the node indicated with a green circle, allowing the remaining node times to be inferred from the genetic data. (*c*) Root-to-tip distances computed from a phylogram, plotted against the ages of the sequences. A regression line is fitted through these data points, with the slope of the line giving an estimate of the evolutionary rate. This method is often used in analyses of time-structured sequence data, such as those from rapidly evolving viruses.

Molecular clocks are based on the assumption that genetic change can be described as a simple function of time (fig. 1). An ideal molecular clock has a number of features: rate constancy through time, rate homogeneity across lineages, taxonomic breadth and applicability, and accessibility of the data. Characters that have evolved at a relatively constant rate are the most suitable for molecular clocks. However, rates of evolution are influenced by a range of biological and extrinsic factors, such as generation length and the efficiency of DNA repair (Bromham 2009). Current phylogenetic methods are able to handle variation in evolutionary rates (Ho and Duchêne 2014), but some forms of rate heterogeneity might be difficult to take into account when using the available models (Dornburg et al. 2012).

Any particular molecular clock is unlikely to be reliable across a broad range of timeframes. For example, some genomic characters are so mutable that they cannot be compared between species. This might make them useful for studying the evolutionary process at the population scale, but it places strong limits on their taxonomic scope. The presence of intraspecific polymorphism, for example, can introduce considerable noise into the dating analysis, particularly when using methods based on linear regression (Lynch and Jarrell 1993; Ehrich et al. 2009). To provide an ideal molecular clock, the data should evolve at a rate that is appropriate for the timescale of the biological events that are being investigated.

## The Past, Present, and Future Diversity of Molecular Clocks

**Fig. 2.—:**
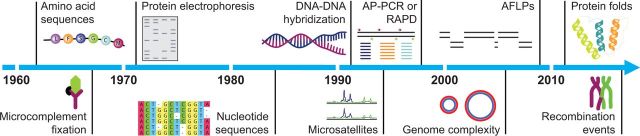
Timeline showing the use of different genetic data types for molecular-clock analyses. The development and analysis of different data types is illustrated by a range of studies over the past five decades: amino acid sequences (Zuckerkandl and Pauling 1962), microcomplement fixation (Sarich and Wilson 1967b), protein electrophoresis (Nei 1971), nucleotide sequences (Miyata and Yasunaga 1980; Cohn et al. 1984), DNA–DNA hybridization (Wayne et al. 1991), microsatellites (Goldstein et al. 1995), randomly amplified polymorphic DNA (Espinasa and Borowsky 1998), genome complexity (Sharov 2006), amplified fragment length polymorphisms (Kropf et al. 2009), protein folds (Wang et al. 2011), and recombination events (Moorjani et al. 2016).

Prior to the wide availability of DNA and protein sequences, a range of biochemical techniques were used to estimate genetic distances between pairs of taxa (fig. 2). Early investigations used microcomplement fixation to examine serum albumin similarity between organisms (Sarich and Wilson 1967a). Subsequently, other biochemical methods were explored, including protein electrophoresis and DNA hybridization (Wayne et al. 1991). These methods are generally effective only for studying closely related taxa (Wilson et al. 1977), so that their utility in molecular dating is usually limited to timescales of a few million years. Biochemical techniques are now rarely used for molecular dating, because they are labor-intensive and offer low resolving power.

Even after DNA sequencing became widespread, the cost of obtaining large data sets long remained prohibitive to most research groups. This was particularly the case in studies of intraspecific evolutionary timescales, for which large sections of sequence would be needed to capture a sufficient amount of variation for estimating genetic distances. This led to the development of molecular clocks based on data from reduced genome representations. For example, amplified fragment length polymorphisms (AFLPs) are coded from sections of DNA generated using restriction enzymes and amplified using PCR. AFLPs have been used to provide a shallow-time molecular clock in several species of alpine plants (Kropf et al. 2009; but see Ehrich et al. 2009) and perciform fishes (Smith et al. 2011). Random amplified polymorphic DNA (RAPD) uses a number of arbitrary primers to amplify anonymous DNA fragments from a genome. A study of primates, antelopes, and *Drosophila* found that genetic distances based on RAPD gel bands were proportional to time since divergence, suggesting some degree of rate homogeneity across lineages (Espinasa and Borowsky 1998). These methods have now largely been superseded by reduced-representation sequencing, which combines the advantages of reduced genome representation and high-throughput sequencing (Davey et al. 2011).

Genome complexity, measured by the size of the nonredundant functional genome, has been proposed to exhibit clocklike evolution (Sharov 2006). This “complexity clock” was constructed using point estimates of the functional genome sizes of mammals, fish, eukaryotes, and prokaryotes, at their approximate divergence times. The functional genome was found to undergo a 7.8-fold increase in size every billion years (Sharov 2006). However, the underlying trend of increasing complexity is only present when considering a directional evolutionary process from prokaryotes to mammals. Perhaps in acknowledgment of this weakness, there has not been any further development of the genomic complexity clock.

Looking towards the future, other features of the genome offer more promising sources of data for molecular clocks. There have been several dating studies that have focused on short tandem repeats of DNA sequences, or microsatellites. Various distance metrics have been proposed for microsatellite data, allowing them to be used in molecular dating based on linear regression (Goldstein et al. 1995; Zhivotovsky 2001). More recently, the development of models of microsatellite evolution has enabled these data to be analyzed using phylogenetic dating methods (Wu and Drummond 2011). There is some evidence that microsatellites evolve at a constant rate within species, including humans and chimpanzees (Sun et al. 2009). In humans, this rate is several orders of magnitude greater than that seen in nucleotides (Sun et al. 2012). Thus, microsatellites might be particularly useful for resolving short evolutionary timescales, especially when large amounts of sequence data would otherwise be needed to present appreciable genetic variation.

When looking at the timescales of ancient evolutionary events, such as the deep divergences in archaea, bacteria, and eukaryotes, nucleotide and amino acid sequences are often saturated with substitutions and cannot be aligned with confidence (Moreira and Philippe 2000). However, protein structure is more conserved than most other genomic characters (Caetano-Anollés et al. 2009). Protein folds have been gained and lost during evolution to enable the development of certain functions, such as aerobic metabolism. These folds have been suggested to arise at a constant rate across all life, forming the basis of a protein-fold clock (Wang et al. 2011). This universal clock has been used to investigate the impact of oxygenation on the early diversification of life (Wang et al. 2011). The protein-fold clock depends on broad sampling of proteomes across taxa, along with reliable prediction of folds, but it has considerable potential for investigating deep evolutionary timeframes.

Recombination events were recently used to estimate the ages of ancient human genomes (Moorjani et al. 2016). If two populations or species shared recent ancestry at a known point in time, the decay of this ancestry through meiotic recombination can be modeled to enable inferences about demographic timescales. Moorjani et al. (2016) used the shared event of Neanderthal admixture as a reference point and examined the difference in accumulated recombination events between ancient and modern genomes. They inferred the ages of five ancient human samples from the Upper Paleolithic, with these estimates showing a strong correlation with the radiocarbon dates of the samples (Moorjani et al. 2016). A key drawback of the recombination clock is that the timing of past admixture events can be difficult to estimate with precision (Sankararaman et al. 2012). However, it offers a useful tool for estimating the ages of ancient samples, particularly when they are beyond the 50,000-year reach of radiocarbon dating (Moorjani et al. 2016). In addition, the recombination clock can be used to analyze data from whole genomes, whereas sequence-based approaches are typically only able to handle linked loci (Shapiro et al. 2011).

## Which Clocks Are the Best Evolutionary Timekeepers?

Various features of the genome can potentially be used for molecular dating, though they are likely to differ in their ability to keep evolutionary time. There have been few direct comparisons of the performance of different molecular clocks (Wayne et al. 1991), but some are clearly most suitable for studies of intraspecific processes, whereas others are only effective for analyzing deep evolutionary events. In many cases, date estimates from other genomic features can be used to validate or complement those obtained from analyses of sequence data or from radiometric methods.

When comparing different types of data for molecular clocks, an important consideration is whether an explicit model of evolution is available. For example, models have been developed for nucleotide sequences, amino acid sequences, binary characters, and microsatellites (Wu and Drummond 2011). Explicit evolutionary models allow the data to be analyzed using statistical phylogenetic approaches (fig. 1*b*), which are likely to be superior to those based on linear regression (fig. 1*a* and *c*). There are two main reasons for this. First, the data points used in the latter are often nonindependent because they have various degrees of shared phylogenetic history (Lynch and Jarrell 1993). A second problem with methods based on linear regression is that they are sensitive to rate heterogeneity across lineages (Duchêne et al. 2016, forthcoming). In contrast, relaxed-clock models can be used to account for among-lineage rate heterogeneity in phylogenetic methods for molecular dating (Ho and Duchêne 2014).

The rapid growth in genomic data opens up unprecedented opportunities for evolutionary analyses. Although molecular dating is almost invariably performed using DNA sequences, genomes offer a rich variety of data that can be used to construct molecular clocks. These other sources of data provide an exciting and potentially valuable avenue of research, particularly in cases where molecular clocks based on DNA sequences might be expected to fail.
